# Baicalein increases the expression and reciprocal interplay of RUNX3 and FOXO3a through crosstalk of AMPKα and MEK/ERK1/2 signaling pathways in human non-small cell lung cancer cells

**DOI:** 10.1186/s13046-015-0160-7

**Published:** 2015-05-07

**Authors:** Fang Zheng, Jingjing Wu, Shunyu Zhao, Qingmei Luo, Qing Tang, LiJun Yang, Liuning Li, WanYing Wu, Swei Sunny Hann

**Affiliations:** Laboratory of Tumor Biology, Guangdong Provincial Hospital of Chinese Medicine, The Second Clinical Medical Collage, University of Guangzhou Traditional Chinese Medicine, Guangzhou, Guangdong Province 510120 China; Department of Medical Oncology, Guangdong Provincial Hospital of Chinese Medicine, The Second Clinical Medical Collage, University of Guangzhou Traditional Chinese Medicine, Guangzhou, Guangdong Province 510120 China; Higher Education Mega Center, No. 55, Neihuan West Road, Panyu District, Guangzhou, Guangdong Province 510006 People’s Republic of China

**Keywords:** Baicalein, NSCLC, RUNX3, FOXO3a, MEK/ERK1/2, AMPKα

## Abstract

**Background:**

Baicalein, a natural flavonoid obtained from the Scutellaria baicalensis root, has been reported to inhibit growth of human lung cancer. However, the detailed mechanism underlying this has not been well elucidated.

**Methods:**

Cell viability was measured using a 3-(4, 5-dimethylthiazol-2-yl)-2, 5-diphenyltetrazolium bromide (MTT) assays. Apoptosis was detected by flow cytometry analysis and caspase 3/7 assays. The expression of RUNX3 and FOXO3a mRNA were measured by real time RT-PCR methods. Western blot analysis was performed to measure the phosphorylation and protein expression of AMP-activated protein kinase alpha (AMPKα) and extracellular signal-regulated kinase 1/2 (ERK1/2), runt-related transcription factor 3 (RUNX3) and forkhead box O3a (FOXO3a). Silencing of FOXO3a and RUNX3 were performed by small interfering RNA (siRNA) methods. Exogenous expression of FOXO3a or RUNX3 was carried out by electroporated transfection assays.

**Results:**

We showed that baicalein significantly inhibited growth and induced apoptosis of non-small cell lung cancer (NSCLC) cells in a time- and dose-dependent manner. Baicalein induced RUNX3 and FOXO3a protein expression, and increased phosphorylation of AMPKα and ERK1/2. Moreover, the inhibitors of AMPK and MEK/ERK1/2 reversed the effect of baicalein on RUNX3 and FOXO3a protein expression. Interestingly, while compound C had little effect on blockade of baicalein-induced phosphorylation of ERK1/2, PD98059 significantly abrogated baicalein-induced phosphorylation of AMPKα. Intriguingly, while silencing of RUNX3 abolished the effect of baicalein on expression of FOXO3a and apoptosis, silencing of FOXO3a significantly attenuated baicalein-reduced cell proliferation. On the contrary, overexpression of FOXO3a restored the effect of baicalein on cell growth inhibition in cells silencing of endogenous FOXO3a gene and enhanced the effect of baicalein on RUNX3 protein expression. Finally, exogenous expression of RUNX3 increased FOXO3a protein and strengthened baicalein-induced phosphorylation of ERK1/2.

**Conclusion:**

Collectively, our results show that baicalein inhibits growth and induces apoptosis of NSCLC cells through AMPKα- and MEK/ERK1/2-mediated increase and interaction of FOXO3a and RUNX3 protein. The crosstalk between AMPKα and MEK/ERK1/2 signaling pathways, and the reciprocal interplay of FOXO3a and RUNX3 converge on the overall response of baicalein. This study reveals a novel mechanism for regulating FOXO3a and RUNX3 signaling axis in response to baicalein and suggests a new strategy for NSCLC associated targeted therapy.

## Introduction

Lung cancer remains one of the leading causes of cancer-related death in men and women worldwide [1]. Non-small cell lung cancer (NSCLC) is more common and the majority of patients present with advanced stage [2]. Emerging data demonstrate promising outcome of other non-surgical treatment in patients with advanced lung cancer. Traditional Chinese medicine (TCM) plays an important role in protecting cancer patients against suffering from other treatment related complications, helping in supportive and palliative care by reducing toxicity of conventional therapy and improving quality of life [3-6]. However, the mechanisms by which TCM in improving the therapeutic efficiency against the lung malignancies remains poorly understood.

Phytochemicals are naturally occurring, plant-based substances that have garnered attention for their anti-cancer properties, both as therapeutics and components of the diet for chemoprevention. One particularly ubiquitous group of phytochemicals is the polyphenolic flavonoids. Baicalein, a natural flavonoid obtained from the Scutellaria baicalensis root, were showed to inhibit proliferation of several malignant tumors including lung cancer [7-10]. One study showed that the therapeutic effects of baicalein are attributed to control proliferation, metastasis and inflammatory microenvironment in human lung cancer cells [7]. Multiple signaling pathways and potential targets involved in the baicalein-suppressed cancer cell growth, including lung, have been reported in the past [7,9-11]. However, the underlying molecular mechanisms associated with its efficacy in targeting lung cancer are largely unknown.

Mammalian forkhead members of the class O (FOXO) transcription factors, a superfamily of proteins are implicated in the regulation of variety of biological functions, such as apoptosis, cell cycle transitions, DNA repair, metabolism, oxidative stress, and cell differentiation [12]. In humans, four members of the FOXO transcription factors (FOXO1, FOXO3a, FOXO4 and FOXO6) have been found [13]; they share a high degree of conserved 100-residue DNA-binding domain, so-called forkhead domain in their DNA-binding area [14]. Among them, FOXO3a has been extensively studied as a crucial protein. Previous studies showed that FOXO3a regulated expression of genes involved in apoptosis, cell cycle arrest, oxidative stress resistance and was negatively regulated by growth factors [15]. During tumor development, inhibition of FOXO3a stimulated cell transformation, tumor progression, and angiogenesis [16]. On the contrary, overexpression of FOXO3a suppressed cancer cell growth, modulated expression of downstream effectors, induced apoptosis, and reduced tumor size [17-19]. These results indicated a tumor suppressor role of FOXO3a, which could be a potential target for the treatment of cancers.

The runt-related transcription factors (RUNXs) belong to a family of conserved proteins, which share the highly homologous DNA-binding, N-terminal Runt domain [20]. To date, three RUNX transcription factors, *i.e.*, RUNX1, RUNX2 and RUNX3, have been identified. Among these, RUNX3, the smallest member of RUNX family, reported to be involved in various cancer processes, such as cell growth, apoptosis, angiogenesis, and metastasis [21]. Study demonstrated that RUNX3 is a tumor suppressor gene, which is absent or mutated in several types of cancers including lung due to hemizygous deletions or epigenetic alterations [22]. One report found that RUNX3 inactivation is a crucial early step in the development of lung malignancy [23].

In this study, we explore the potential mechanism by which baicalein controls lung cancer cell proliferation.

## Materials and methods

### Reagents

Monoclonal antibodies against to total ERK1/2, AMPKα and the phosphor-forms were purchased from Cell Signaling Technology Inc. (Beverly, MA, USA). The FOXO3a and RUNX3 antibodies were obtained from Epitomics (Burlingame, CA, USA). PD98059 (MAPK extracellular signaling-regulated kinase (ERK) kinase (MEK)/ERK1/2 inhibitor) and compound C (inhibitor of AMPK) were purchased from Merck Millipore (Billerica, MA, USA), MTT powder was purchased from Sigma Aldrich (St. Louis, MO, USA). FOXO3a and RUNX3 small interfering RNAs (siRNAs) were obtained from Santa Cruz Biotechnology, Inc. (Santa Cruz, CA, USA). Baicalein was purchased from Chengdu Must Bio-technology Company (Chengdu, Sichuan, China). The drugs were freshly diluted to the final concentration with culture medium before applying to experiments.

### Cell lines and cultures

Human lung adenocarcinoma cells (PC9, H1299, H1650, A549, H358 and H1975) were obtained from the Chinese Academy of Sciences Cell Bank of Type Culture Collection (Shanghai, China) and the Cell Line Bank at the Laboratory Animal Center of Sun Yat-sen University (Guangzhou, China). The cells were cultured at 37°C in a humidified atmosphere containing 5% CO2. The culture medium consisted of RPMI 1640 medium obtained from GIBCO, Life Technologies (Grand Island, NY, USA) supplemented with 10% (v/v) heat-inactivated fetal bovine serum (Thermo Fisher Scientific Inc, Waltham, MA, USA), 100 μg/ml streptomycin and 100 U/mL penicillin. When cells reached 75% confluence, they were digested with 0.25% trypsin for passage for the following experiments.

### Cell viability assay

We used 3-(4, 5-dimethylthiazol-2-yl)-2, 5-diphenyltetrazolium bromide (MTT) method to determine cell viability as described previously [19]. Briefly, NSCLC cells were harvested, counted and seeded into a 96-well microtiterplate, 5 × 10^3^ cells/well. The cells were treated with increasing concentrations of baicalein for up to 72 h. After incubation, 10 μL MTT solution (5 g/L) was included to each well and cells were incubated at 37°C for an additional 4 h, followed by removing the supernatant, adding 150 μL solvent dimethyl sulfoxide (DMSO), and oscillating for 10 min. Afterwards, absorbance at 570 nm was determined through the use of ELISA reader (Perkin Elmer, Victor X5, Waltham, MA, USA). Each experiment was repeated three times. Cell viability (%) was calculated as follows: (absorbance of test sample/absorbance of control) × 100%.

### Cell apoptosis assays

Annexin V-FITC/PI Apoptosis Detection Kit (BD Biosciences, San Jose, CA, USA) was used to detect cell apoptosis according to instructions from the manufacturer. Briefly, after treated with baicalein for 24 h, the apoptotic cells were harvested by Trypsin (no EDTA) and washed with phosphate-buffered saline (PBS), then resuspended the cells in 500 μL binding buffer, 5 μL Annexin V-FITC regent and 10 μL PI regents and incubated for 5 min at room temperature (RT) in the dark, followed by detecting cell apoptosis by Flow cytometry (FC500, Beckman, USA).

### Detection of caspase-3/7 activity

We examined the activity of caspase-3/7 using the Caspase-Glo 3/7 Assay kit (Promega, Madison, WI, USA), which based on the manufacturer’s instruction. Briefly, NSCLC cells were seeded in 96-well plates and treated with or without baicalein for 48 h. Afterwards, the cells were lysed and incubated with 100 μL of Apo-ONE Caspase-3/7 reagent (substrate and buffer in the ratio of 1:100). After 1 h incubation in the dark at RT, the fluorescence of each well was measured at 485–520 nm by reading in an Epoch microplate reader (Biotek Instruments; Winooski, VT, USA).

### Quantitative real-time PCR (qRT-PCR)

A quantitative real-time RT-PCR assay was developed for the detection and quantification of RUNX3 and FOXO3a transcripts using GAPDH as an endogenous control. The primers used in this study were designed as follows: RUNX3 forward 5’- 5′-TTATGAGGGGTGGTTG-TATGTGGG-3′ and reverse 5′-AAAACAACC AACACAAACACCTCC-3′ [24]. FOXO3a and GAPDH (used as an internal control) used the following primers: forward 5′-GCAAGCACAGAGTTGGATGA-3′ *(*F) and reverse 5′-CAGGTCGTCCATGAGGTTTT -3′(R) for FOXO3a [25] and forward 5’- AAGCCTGCCGGTGACTAAC -3’; reverse 5’- GCGCCCAATACGACCAAATC -3’ for GAPDH. Total RNA was extracted using the TRIzol solution and the first-strand cDNA was synthesized from total RNA (2 μg) by reverse transcription using oligo-dT primers and Superscript II reverse transcriptase (Invitrogen, Grand Island, NY, USA) according to the manufacturer’s instructions. Quantitative real-time PCR was performed in a 20 μL mixture containing 2 μL of the cDNA preparation, 10 μL 2X SYBR Green Premix ExTaq, and 10 μM primer on an ABI 7500 Real-Time PCR System (Applied Biosystems, Grand Island, NY, USA). The PCR conditions were as follows: 0.5 min at 95°C, followed by 40 cycles of 5 s at 95°C, and 34 s at 60°C. Each sample was tested in triplicate. Threshold values were determined for each sample/primer pair; the average and standard errors were calculated. The relative expression levels of the target genes RUNX3 and FOXO3a were normalized to that of GAPDH. The data were analyzed using the comparative threshold cycle (2^−ΔΔCT^) method.

### Western blot analysis

The detailed method was based on previous report [19]. After measuring the protein concentrations using the Bio-Rad protein method, whole cell lysates containing same amount of protein were solubilized in 4× SDS-sample buffer and separated on 10% SDS polyacrylamide gels. Membranes (Millipore, Billerica, MA, USA) were incubated with antibodies against ERK1/2, AMPKa, pERK1/2, p-AMPKα, FOXO3a and RUNX3 (1:1000). The membranes were washed and incubated with a secondary goat antibody raised against rabbit IgG conjugated to horseradish peroxidase (Cell Signaling, Beverly, MA, USA). The membranes were washed again and transferred to freshly made ECL solution (Immobilon Western; Millipore, Billerica, MA, USA) and observed, recorded the signals using the Gel Imagine System (Bio-Rad, Hercules, CA, USA) or exposed to X-ray film.

### Treatment with FOXO3a and RUNX3 small interfering RNAs (siRNAs)

For the transfection procedure, cells were seeded in 6-well or 96-well culture plates in RPMI 1640 medium containing 5% FBS (no antibodies), grown to 70% confluence, and FOXO3a. RUNX3 and control siRNAs were transfected using the lipofectamine 2000 reagent according to the manufacturer’s instructions. Briefly, Lipofectamine 2000 was incubated with Opti-MEM medium (Invitrogen, Carlsbad, CA, USA) for 5 min, mixed with siRNA (up to 50 nM), and incubated for 20 min at room temperature before the mixture was added into the cells. After culturing for up to 30 h, the cells were washed and resuspended in fresh media in the presence or absence of baicalein for an additional 24 h for all other experiments.

### Electroporated transfection assays

The detailed procedure was based on the protocol from the provider (Bio-Rad, Hercules, CA, USA). Briefly, NSCLC cells (5x10^7^ cells/mL) were transferred into conical tubes and centrifuged at 1200 rpm for 5 min. After centrifuging, the medium were removed and the cells were washed with 1X PBS, and centrifuged again at 1200 rpm for 5 min. Afterwards, the tubes were added Bio-Rad Gene Pulser electroporation buffer. After resuspending the cells, the desired N1-GFP or FOXO3a-GFP plasmid DNA, kindly provided Frank M. J. Jacobs (Rudolf Magnus Institute of Neuroscience, Department of Pharmacology and Anatomy, University Medical Center, Utrecht, Netherlands) and was reported previously [26] and control (pCMV-6) or RUNX3 expression vector (RUNX3-pCMV6-AC-GFP, obtained from OriGene Technologies, Inc. Rockville, MD, USA) at a final concentration of 10 μg/mL were added and the electroporation plate were put in the MXcell plate chamber and closed the lid. The electroporation conditions on the plates to deliver 150 V/5 ms square wave were adjusted until reaching the optimum. After electroporation was completed, the cells were transferred to a tissue culture plate. We typically transfer each 150 μL electroporation sample to a 6-well tissue culture plate containing 2 mL RPMI1640. Cells were incubated 48 h at 37°C, then treated with baicalein for an additional 24 h.

### Statistical analysis

All experiments were repeated a minimum of three times. All data are expressed as mean ± SD. Differences between groups were assessed by one-way ANOVA and significance of difference between particular treatment groups was analyzed using Dunnett’s multiple comparison tests or Bonferroni t-test using GraphPad Prism software version 5.0 (GraphPad Software, Inc. La Jolla, CA , USA). Asterisks showed in the figures indicate significant differences of experimental groups in comparison with the corresponding control condition. P-values <0.05 were considered statistically significant.

## Results

### Baicalein inhibited growth and induced apoptosis of human NSCLC cells in a dose- and time-dependent manner

**Figure 1:**
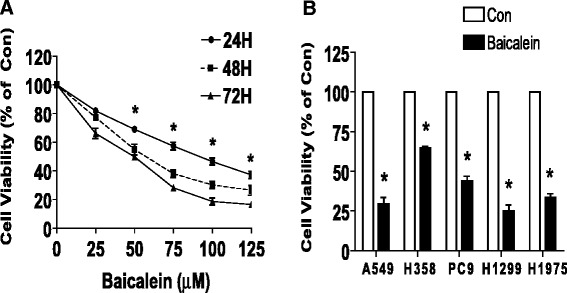
Baicalein inhibited growth of human lung carcinoma cells in a dose- and time-dependent manner. **A**, H1650 cells were treated with increased concentrations of baicalein for up to 72 h to examine the cell viability by MTT assays as described in the Materials and Methods section. **B**, NSCLC cell lines indicated were treated with baicalein (75 μM) for 48 h. The cell viability was determined using the MTT assay and was expressed as percentage of control in the mean ± SD of three separate experiments. *indicates significant difference as compared to the untreated control group (P < 0.05).

We first detected the effect of baicalein on cell growth in human NSCLC cells H1650 by MTT assay. As show in Figure 1A, baicalein decreased the cell viability in a dose- and time-dependent manner with maximal dose of 75 μM at 48 h treatment. Similar results were also observed in other NSCLC cell lines (Figure 1B).

**Figure 2:**
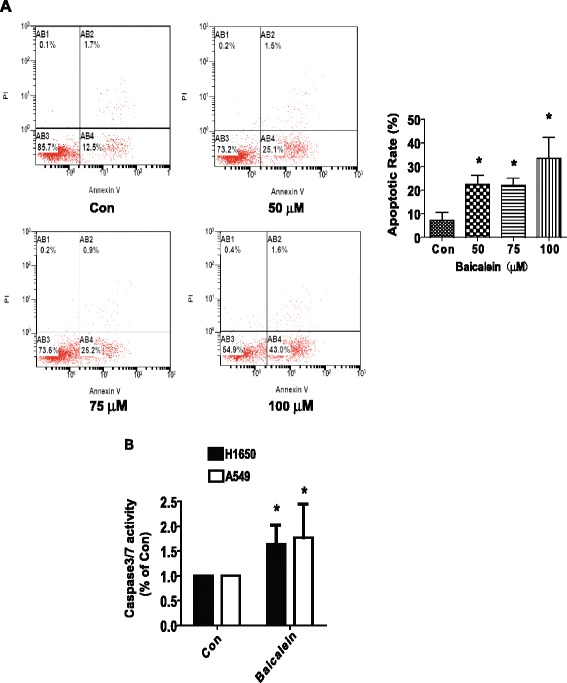
Baicalein induced apoptosis in NSCLC cells. **A**, H1650 cells were treated with increased concentrations of baicalein for 24 h. Afterwards, cells were harvested for analysis of apoptosis using the Annexin V-FITC/PI Apoptosis Detection Kit as detailed in Materials and Methods section. The AB3 quardrant (annexin V-/PI-), AB4 quadrant (annexin V+/PI-) and AB2 quadrant (annexin V+/PI+) of the histograms indicated the percentage of normal cells, early apoptosis and late apoptosis, respectively. Data are expressed as a percentage of total cells. Values in bar graphs were given as the mean ± SD from three independent experiments performed in triplicate. *indicates significant difference as compared to the untreated control group (P < 0.05). **B**, Caspase 3/7 activity assay was performed on H1650 and A549 cells treated with or without baicalein for 48 h. Relative caspase 3/7 activity is indicated as percentage of untreated control cells. Results represent those obtained in three experiments. *indicates significant difference as compared to the untreated control group (P < 0.05). **indicates significant difference from baicalein treated alone (P < 0.05).

We also examined the effect of baicalein on apoptosis in NSCLC cells. We found that H1650 cells treated with increased concentrations of baicalein for 24 h resulted in induction of apoptosis shown in the lower right (AB4) quadrants of the histograms, which were counted as “early” apoptotic cells (Figure 2A) as detected using the Annexin V-FITC/PI stain Apoptotic Detection Kit. After 24 h of treatment, the baicalein-induced apoptotic rate was greater than that in the non-treated control cells (Figure 2A). Similar results were obtained in an additional NSCLC cell line A549 cells (not shown). Meanwhile, the effect of baicalein on apoptosis of H1650 and A549 cells was also tested by measuring enzymatic caspase 3/7 activity. We observed the increased caspase 3/7 activity by baicalein (Figure 2B). The above results indicated that baicalein induced apoptosis in NSCLC cells.

### Baicalein increased the phosphorylation of ERK1/2 and AMPKα in a time-dependent manner

**Figure 3:**
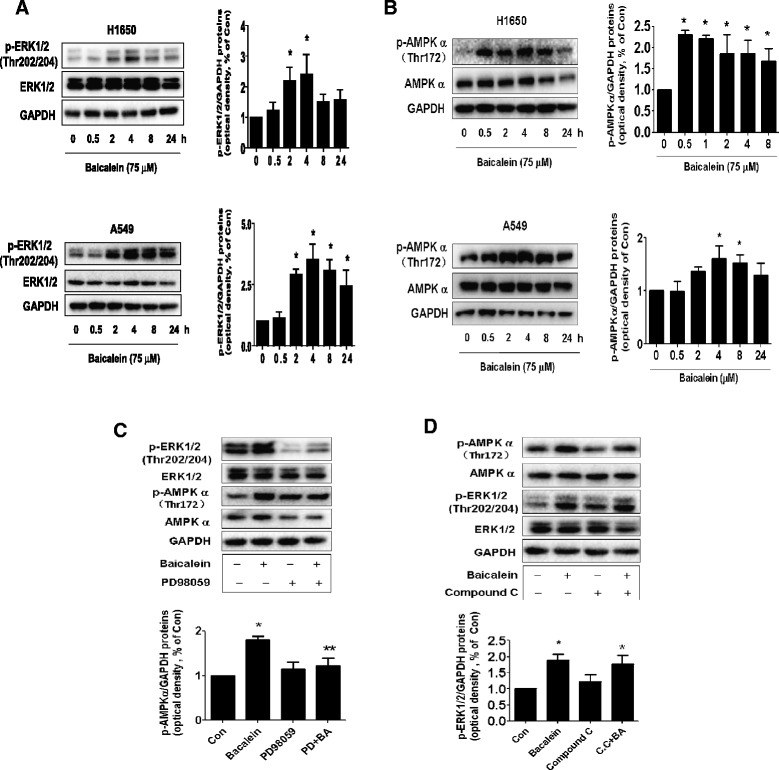
Baicalein increased the phosphorylation of ERK1/2 and AMPKα in a time-dependent manner. **A-B**, A549 and H1650 cells were treated with baicalein (75 μM) in the indicated times, and cell lysate was harvested and the expression of the phosphorylated or total protein of ERK1/2, AMPKα were measured by Western blot analysis using corresponding antibodies. GAPDH was used as loading control. **C-D**, H1650 cells were treated with PD98059 (10 μM) **(C)** or compound C (C.C, 5 μM) **(D)** for 2 h before exposure of the cells to baicalein (BA, 75 μM) for an additional 24 h. Afterwards, the expression of p-ERK1/2 and p-AMPKα protein and their total forms was detected by Western blot. The bar graphs represented the densitometry results of p-ERK or AMPKα /GAPDH as mean ± SD of at least three separate experiments. *indicates significant difference from untreated control cells (P < 0.05). **indicates significant difference from baicalein treated alone (P < 0.05).

MEK/ERK1/2 and AMPK signaling pathways were involved in apoptosis and cell growth depending on the cell types and stimulus. We showed that baicalein increased the phosphorylation of ERK1/2 and AMPKα in a time-dependent manner in H1650 and A549 cells (Figure 3A-B). Note that the expression of total ERK1/2 and AMPKα proteins had no significant changes after baicalein treatment. The reported data demonstrated a cross-talk between MEK/ERK and AMPK pathways in other system [27,28]. In this study, we found that, while an AMPK inhibitor, compound C, had little effect on influencing baicalein-increased the phosphorylation of ERK1/2, the inhibitor of MEK/ERK1/2 (PD98059), eliminated the baicalein-induced phosphorylation of AMPKα (Figure 3C-D). The latter was also observed using another selective MEK/ERK1/2 inhibitor (U0126) (not shown). This result demonstrated that activation of MEK/ERK1/2 led to stimulation of AMPK signaling in this process.

### Baicalein increased protein expression of FOXO3a and RUNX3 through ERK1/2 and AMPK pathways

**Figure 4:**
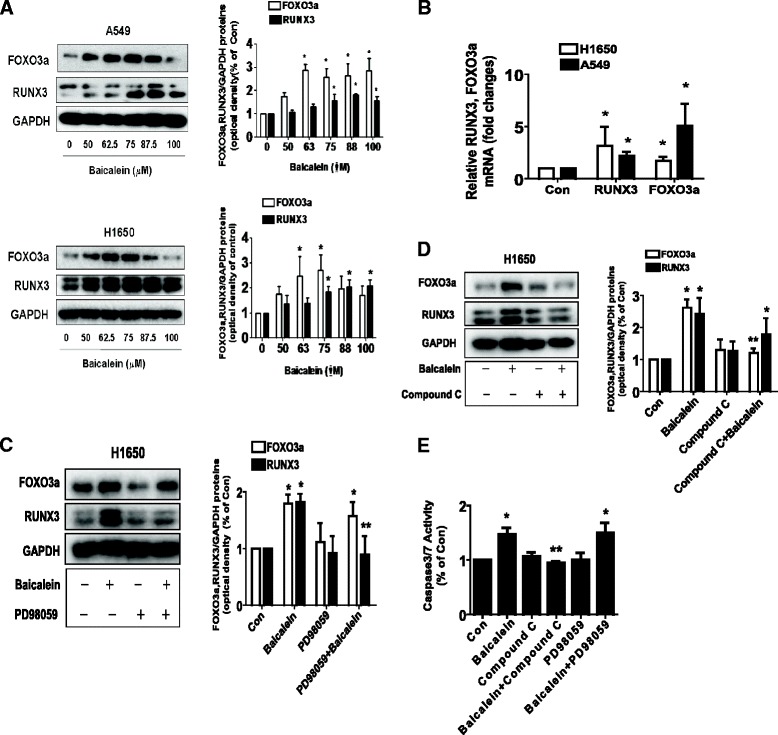
Baicalein increased protein levels of FOXO3a and RUNX3 through ERK1/2 and AMPK pathways. **A**, H1650 and A549 cells were exposed to increased concentration of baicalein for 24 h. Afterwards, the expression of FOXO3a and RUNX3 protein were detected by Western blot. **B**, H1650 and A549 cells were exposed to baicalein for 24 h. Afterwards, the expression of FOXO3a and RUNX3 mRNAs was detected by real-time RT-PCR method as described in the Materials and Methods section. **C-D**, H1650 cells were treated with PD98059 (10 μM) **(C)** or compound C (5 μM) **(D)** for 2 h before exposure of the cells to baicalein (75 μM) for an additional 24 h. Afterwards, the expression of FOXO3a and RUNX3 protein were detected by Western blot using antibodies against FOXO3a and RUNX3. The bar graphs represent the mean ± SD of RUNX3/GAPDH and FOXO3a/GAPDH of three independent experiments. **E**, H1650 cells were treated with PD98059 (10 μM) or compound C (5 μM) for 2 h before exposure of the cells to baicalein (75 μM) for an additional 24 h. Afterwards, the cells were collected and processed for analysis of apoptosis as determined by caspase 3/7 activity assays. Results represent those obtained in three experiments. *indicates significant difference as compared to the untreated control group (P < 0.05). **indicates significant difference from baicalein treated alone (P < 0.05).

In this study, we showed that baicalein increased FOXO3a, a transcription factor with known anti-tumor activity [29] and RUNX3, another tumor suppressor, protein expression in a dose-dependent fashion in A549 and H1650 cells (Figure 4A). As expected, baicalein increased FOXO3a and RUNX3 mRNA levels as determined by qRT-PCR methods (Figure 4B) in A549 and H1650 cells. Next, we used specific inhibitors of MEK/ERK1/2 and AMPK to pre-treated H1650 cells to examine the role of these kinase in mediating the effect of baicalein on induction of FOXO3a and RUNX3. As shown in Figure 4C and D, the inhibitors of MEK/ERK1/2 (PD98059) and AMPK (compound C) either in part or completely abolished baicalein-induced RUNX3 and FOXO3a protein expression (Figure 4C-D). This result showed that activation of MEK/ERK1/2 and AMPK was involved in the baicalein-induced protein expression of RUNX3 and FOXO3a.

Previously, we showed that baicalein induced apoptosis in lung cancer cells. To further examine the role of ERK1/2 and AMPK signaling pathways in this process, we first treated cells with MEK/ERK1/2 and AMPK inhibitors before exposing the cells to baicalein. As shown in Figure 4D, compared with baicalein treated alone, compound C blocked the baicalein-induced caspase 3/7 activity, while PD98059 had no such effect. This result indicated the role of activation of AMPK but not MEK/ERK1/2 in mediating the effect of baicalein on induction of apoptosis in NSCLC cells.

### Induction of FOXO3a and RUNX3 involved in baicalein-inhibited cell growth and apoptosis

**Figure 5:**
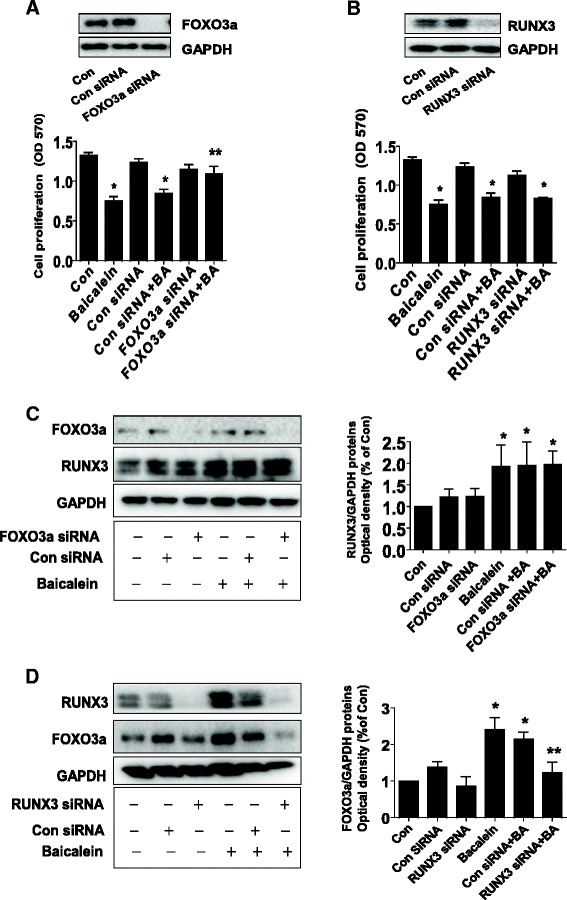
Induction of FOXO3a and RUNX3 mediated baicalein-inhibited cell growth. **A-B**, H1650 cells were transfected with control or FOXO3a, or RUNX3 siRNAs with lipofectamine 2000 reagent for 24 h, followed by exposure the cells to baicalein (BA, 75 μM) for an additional 24 h. Afterwards, the cells proliferation was detected using MTT assays. The expression of FOXO3a and RUNX3 protein was determined by Western blot. The bar graphs represent the mean ± SD of RUNX3/GAPDH and FOXO3a/GAPDH of three independent experiments. The insert blots represent the protein expression of FOXO3a and RUNX3. **C-D**, H1650 cells were transfected with control or FOXO3a or RUNX3 siRNAs (50 nM each) for 24 h before exposing the cell to baicalein (BA) for an additional 24 h. Afterwards, the expression of FOXO3a and RUNX3 protein was determined by Western blot. *indicates significant difference from untreated control cells **indicates significant difference from baicalein treated alone (P < 0.05).

Studies have shown that FOXO3a and RUNX3 regulated cell growth and apoptosis. In this study, we further tested the potential interaction of these molecules that may affect the lung cancer cell growth. We found that silencing of FOXO3a but not RUNX3 by siRNA approaches significantly reversed the baicalein-inhibited lung cancer cell growth (Figure 5A-B). Note that silencing of FOXO3a and RUNX3 largely reduced the FOXO3a and RUNX3 protein expression (Figure 5A-B, upper panel). Interestingly, while silencing of FOXO3a had little effect on influencing baicalein-induced RUNX3 protein expression (Figure 5C), knockdown of RUNX3 significantly attenuated the baicalein-induced protein expression of FOXO3a (Figure 5D).

### Overexpression of FOXO3a and RUNX3 restored cell growth and attenuated apoptosis affected by baicalein

**Figure 6:**
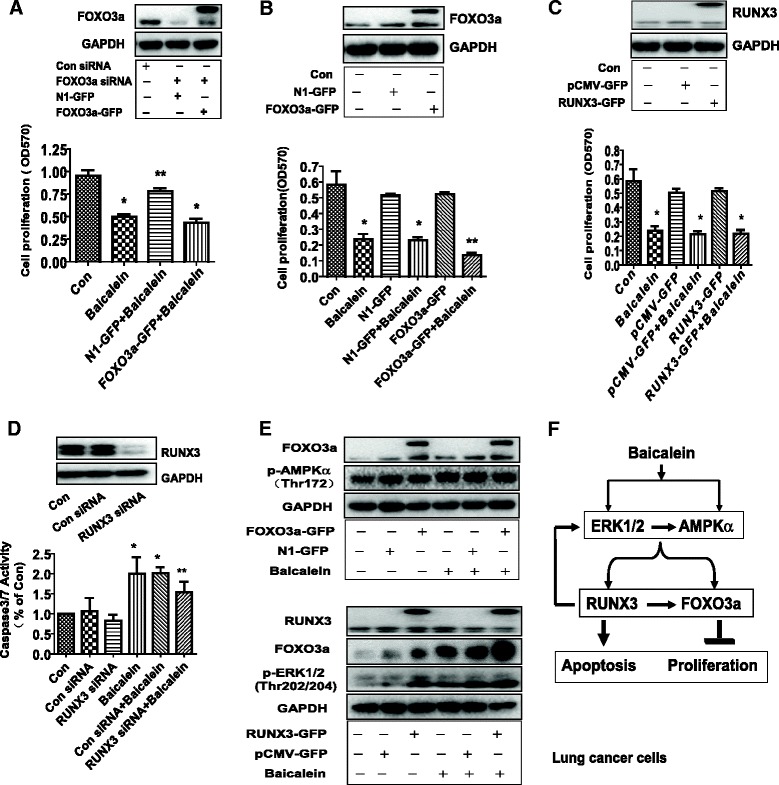
Overexpression of FOXO3a and RUNX3 restored cell growth and attenuated apoptosis affected by baicalein. **A**, H1650 cells were transfected with control or FOXO3a siRNA for 30 h, followed by control or FOXO3a expression vectors for up to 24 h before exposure of the cells to baicalein for an additional 24 h. Afterwards, cell growth was determined by MTT assays. The upper insert panel represents blots of expression of FOXO3a protein detected by Western blot. **B-C**, H1650 cells were transfected with control or FOXO3a, or RUNX3 expression vectors for 24 h before exposing the cells to baicalein for an additional 24 h. Afterwards, cell viability were detected by MTT assays. Insert blots were FOXO3a and RUNX3 protein expression. **D**, H1650 cells were transfected with control or RUNX3 siRNA for 30 h before exposing the cells to baicalein for an additional 24 h. Afterwards, the cells were processed for analysis of apoptosis as determined by caspase 3/7 activity assays. Data are expressed as a percentage of total cells. Values in bar graphs were given as the mean ± SD from three independent experiments. *indicates significant difference as compared to the untreated control group (P<0.05). **indicates significant difference from baicalein treated alone (P<0.05). **E**, H1650 cells were transfected with control or FOXO3a, or RUNX3 expression vectors for 24 h before exposing the cells to baicalein for an additional 2 h. Afterwards, The expression of FOXO3a and RUNX3 protein, phosphorylation of AMPKα and ERK1/2 were determined by Western blot. **F**, The graph shows that baicalein inhibits growth and induces apoptosis of lung cancer cells through AMPKα- and ERK1/2-mediated increase in RUNX3 and FOXO3a protein expression. Overexpression of RUNX3 strengthens baicalein-induced phosphorylation of ERK1/2 and induces expression of FOXO3a. The crosstalk between AMPKα and ERK1/2, and the reciprocal incorporation of FOXO3a and RUNX3 converge on the overall anti-cancer responses of baicalein.

On the contrary, overexpression of FOXO3a restored the effect of baicalein on cell growth in cells silencing of endogenous FOXO3a gene by siRNA methods (Figure 6A). In addition, it also enhanced the effect of baicalein on cell growth inhibition (Figure 6B). Intriguingly, while exogenous expression of RUNX3 had no effect on influencing baicalein-inhibited cell growth (Figure 6C), silencing of RUNX3 by siRNA significantly attenuated the effect of baicalein on caspase 3/7 activity (Figure 6D). The above findings indicated that the induction and potential reciprocal interactions of FOXO3a and RUNX3 contributed to the baicalein-inhibited cell growth and -induced apoptosis. This also implied that the inhibition of proliferation could be in part an outcome of increased cell apoptosis or *vise versa.* Moreover, we showed that, while overexpression of FOXO3a had no further effect on phosphorylation of AMPKα, exogenous expression of RUNX3 strengthened the effect of baicalein on phosphorylation of ERK1/2 (Figure 6E) and induced FOXO3a protein expression (Figure 6E).

## Discussion

Previous studies showed that baicalein could be considered as a potential candidate for the treatment of human cancers. However, the exact mechanisms involving in the effect of baicalein on inhibition of cancer cell growth are not fully understood. In this study, consistent with others [7,8,30], baicalein showed significant cytotoxicity and induced apoptosis in NSCLC cells. The concentrations of baicalein used in this study and demonstrated to inhibit lung cancer cell growth were consistent with other studies, which showed a substantial effect on inhibition of cancer cell growth and induction of apoptosis at physiological doses [9,10,30].

Several signaling pathways and potential targets (genes or/and proteins) that involved in the overall responses of baicalein in inhibition of growth and induction of apoptosis in cancer cells have been reported [9,10,31]. Consistent with this, our results demonstrated that, in addition to ERK1/2, activation of AMPKα signaling was also implicated in the effect of baicalein on induction of FOXO3a and RUNX3 expression. AMPK is the central component of protein kinase cascade that plays a key role in the regulation of energy control. Activated AMPK induces catabolic metabolism and suppresses the anabolic state, thereby inhibiting cancer cell proliferation and serving as a tumor suppressor [32,33]. Our results suggested that activation of MEK/ERK1/2 led to stimulation of AMPKα signaling and the reciprocal interaction of MEK/ERK1/2 and AMPKα signaling pathways contributed to the overall responses of baicalein in the control of lung cancer cell proliferation. The crosstalk between MEK/ERK1/2 and AMPKα signaling in mediating the physiopathological responses of cancer cell survival have been reported in other studies [27,28], demonstrating the critical roles of the complicated signaling networks in regulation of gene expression and cancer cell survival. Nevertheless, more experiments, such as siRNAs and overexpression of the constitutive active form of kinases, are needed to confirm the loss of MEK/ERK1/2 in preventing the activation of AMPK. Of note, recent studies suggested dual roles of AMPK played in cancer biology depending on environmental context [34]. We believed that the truly insight into the role of AMPK in suppressing tumor growth needs to be well characterized.

Our study suggested that increased FOXO3a and RUNX3 expression were involved in the inhibition of NSCLC cell proliferation. FOXO3a is a member of the FOXO transcription factor family, which modulate the expression of genes involved in cell cycle arrest, apoptosis, autophagy, and other cellular processes. We previously demonstrated that induction of FOXO3a was involved in the berberine- and curcumin-, two bioactive components extracted from TCM herbs, inhibited growth and -induced apoptosis in NSCLC and nasopharyngeal carcinoma cells [17,19]. Consistent with this, others also showed similar results indicating the tumor suppressor role of this transcriptional factor [35,36]; this implied that FOXO3a represents an attractive therapeutic target in the chemoprevention and possibly in inhibition of progression of human cancers. In addition, we for the first time demonstrated the inhibitory effect of baicalein on RUNX3, another tumor suppressor whose reduced expression may play an important role in the development and progression of several cancer types including lung [23,37,38]. Inactivation of RUNX3 was a crucial early event in the occurrence and development of lung malignancy [23,39,40], and upregulation of RUNX3 inhibited lung cancer cell growth [41]. Study implicated a central role of RUNX3 downregulation in lung adenocarcinoma occurrence that may be independent of other well-known cancer-related pathways and suggested potential diagnostic implications [22]. This also highlighted a critical role of this tumor suppressor in the treatment of lung cancer. Our results indicated that RUNX3 could be an upstream of FOXO3a and that silencing and overexpression of RUNX3 could regulate the FOXO3a expression, this together with the data from silencing of RUNX3 in refraining apoptosis suggested that the expression and interplay between these two molecules played an important role in influencing the overall responses of baicalein. One study showed that RUNX3 could interact with FOXO3a to induce the expression of pro-apoptotic proteins, thereby triggering apoptosis in gastric cancer cells [42]. Nevertheless, the detailed mechanism of this interplay in mediating anti-tumor activity of baicalein required to be further elucidated.

Intriguingly, our results also suggested the important roles of ERK1/2 and AMPKα signaling pathways in mediating the effect of baicalein on induction of FOXO3a and RUNX3 proteins. Reports from ours and other studies demonstrated that activation of MEK/ERK1/2 or/and AMPK contributed to increase in FOXO3a protein, decrease in cancer cell growth, and other functions in several cell systems [17,43-46]. Novel baicalein derivatives found to activate AMPK in various tumor cell types [45]; moreover, one report showed that, by activation of AMPKα-mediated multiple downstream intracellular signaling pathways, baicalein could protect mice from metabolic syndrome induced by a high-fat diet [46]. Of note, inactivation ERK1/2 signaling was involved in the induction of FOXO3a and RUNX3 in other studies [47,48]. The discrepancy remains unclear; different stimuli, cell lines used and environmental contexts may be responsible for this, which need to be determined in the future studies. Moreover, there were no reports demonstrating the link of AMPK signaling and RUNX3 expression. We believed that our findings provided the novel insight into the connection between AMPKα signaling and expression of RUNX3 affected by baicalein, and also highlighted the tumor suppressor role of AMPKα and RUNX3 that were involved in the anti-tumor effect of baicalein. Furthermore, we for the first time demonstrated a positive feedback regulation of ERK1/2 signaling by RUNX3, in turn, this would further enhance the anti-tumor efficacy of baicalein. This, together with the data showing that silencing of FOXO3a and RUNX3 reversed the effect of baicalein on cell proliferation and apoptosis, confirmed the critical roles of FOXO3a and RUNX3 played in this process. It is possible that inhibition of proliferation can be in part a consequence of increased apoptosis or *vise versa.* We predicted that FOXO3a and RUNX3 could be valuable prognostic markers as well as potential molecular targets for lung cancer. Note that, while baicalein has been shown to increase FOXO3a and RUNX3, whether this was due to a transcriptional (e.g., mRNA expression) or posttranscriptional regulation (e.g., protein stability) required to be determined.

## Conclusion

Collectively, our results show that baicalein inhibits growth and induces apoptosis of NSCLC cells through MEK/ERK1/2- and AMPKα-mediated increase in FOXO3a and RUNX3 proteins, respectively. Overexpression of RUNX3 increases FOXO3a and strengthens baicalein-induced phosphorylation of ERK1/2. The activation and cross-talk of AMPKα and MEK/ERK1/2 signaling pathways, and reciprocal interplay of RUNX3 and FOXO3a expression contribute to the overall responses of baicalein, which unveils a novel molecular mechanism by which baicalein controls human lung cancer cell growth (Figure 6F).

## Abbreviations

MTT: 3-(4, 5)-dimethylthiahiazo (-z-y1)-3, 5-di-phenytetrazoliumromide
AMPKα: AMP-activated protein kinase alpha
TCM: Traditional Chinese medicine
FOXO: Forkhead members of the class O
ERK1/2: Extracellular signal-regulated kinase 1/2
siRNA: Small interfering RNA
NSCLC: Non-small cell lung cancer
FOXO3a: Forkhead box-O 3a
Runx3: Runt-related transcription factor 3
DMSO: Dimethylsulfoxide
PBS: Phosphate-buffered saline
RT: Room temperature
qRT-PCR: Quantitative Real-Time RT-PCR
MEK: MAPK extracellular signaling-regulated kinase (ERK) kinase
